# Comparison of Volatile Anesthesia and Intravenous Anesthesia for Endovascular Thrombectomy in Acute Ischemic Stroke Patients Under General Anesthesia: A Systematic Review and Meta‐Analysis

**DOI:** 10.1155/ccrp/7360200

**Published:** 2026-05-28

**Authors:** Marluce Gois de Oliveira, Wellington de Araujo, Leonardo Saraiva Guimaraes de Oliveira, Livia Kneipp Rodrigues, Lucas Area Leao Barreto, Yara Silva Dias, Nathan Pace, Talmage Egan

**Affiliations:** ^1^ School of Medicine, Pontificia Universidade Catolica do Parana—PUC/PR, Curitiba, Parana, Brazil; ^2^ Department of Neurology, University of Utah—School of Medicine, Salt Lake, Utah, USA; ^3^ Department of Anesthesiology, Faculdade de Ciências Médicas de Minas Gerais, Belo Horizonte, Minas Gerais, Brazil; ^4^ Department of Anesthesiology, Universidade Federal de Minas Gerais, Belo Horizonte, Minas Gerais, Brazil, ufmg.br; ^5^ School of Medicine, Universidade Federal do Ceara, Fortaleza, Ceara, Brazil; ^6^ Department of Anesthesiology, Hospital Sancta Maggiore, Sao Paulo City, Sao Paulo, Brazil; ^7^ Department of Anesthesiology, University of Utah —School of Medicine, Salt Lake, Utah, USA

**Keywords:** acute ischemic stroke, intravenous anesthesia, thrombectomy, volatile anesthesia

## Abstract

**Background:**

Endovascular thrombectomy (EVT) is a well‐established treatment for acute ischemic stroke (AIS). However, the optimal choice of general anesthesia (GA) during this procedure remains uncertain. This study aims to compare the effects of volatile anesthesia (VA) and total intravenous anesthesia (TIVA) on outcomes in AIS patients undergoing EVT.

**Methods:**

We conducted a systematic search of PubMed, Embase, and Cochrane databases for studies comparing VA and TIVA in this context. Key outcomes assessed were favorable functional outcome (defined as a Modified Rankin Scale [mRS] 0–2), mortality, successful recanalization (mTICI 2b/3), and intraoperative mean arterial pressure (MAP). A subgroup analysis was also performed for patients with anterior circulation stroke.

**Results:**

A total of 568 patients from four observational studies were included, of whom 187 (33%) received TIVA. In the unadjusted analysis, no statistically significant differences were observed between TIVA and VA in terms of favorable functional outcome (OR 1.28; *p* = 0.403), mortality (OR 0.54; *p* = 0.082), successful recanalization (OR 1.38; *p* = 0.344), or MAP (MD −0.56; *p* = 0.755). Adjusted ORs (aORs) were extracted from multivariable models, with covariates varying across studies. The aORs suggested TIVA may be associated with improved outcomes, being linked to a higher likelihood of favorable functional outcome (aOR 1.96, 95% CI [1.15–3.34], *p* = 0.013) and reduced mortality (aOR 0.40, 95% CI [0.20–0.79], *p* = 0.008). In the subgroup of patients with anterior circulation stroke, TIVA was similarly associated with better recovery (aOR 2.66; *p* = 0.033) and lower mortality (aOR 0.38; *p* = 0.002) at 3 months, but no significant differences were found when the timing after stroke was not specified.

**Conclusion:**

Among AIS patients undergoing EVT, TIVA may lead to improved functional outcome and reduced mortality compared to VA. These findings support the use of TIVA as a potentially more favorable strategy in this context, warranting further prospective trials.

## 1. Introduction

Stroke is a devastating disorder that affects millions of people around the world, causing disability and death. Numerous drug and surgical trials have been conducted to find effective treatments for these patients [1, 2]. Among these treatments, endovascular thrombectomy (EVT) is recognized as the standard of care for acute ischemic stroke (AIS) due to large vessel occlusion [3, 4] and should not be delayed or precluded by intravenous thrombolytics regardless of its success [5].

Earlier meta‐analyses [1, 2] showed that general anesthesia (GA) is both safer and more effective than conscious sedation during EVT. However, the choice of anesthetic agents in GA had not been previously assessed. This meta‐analysis serves as the first direct comparison between volatile anesthesia (VA) and total intravenous anesthesia (TIVA) during EVT for AIS.

GA is essential in the management of patients undergoing EVT for AIS. It offers various benefits, including reliable airway control, patient immobility, and facilitating the management of apnea and hemodynamic parameters during crucial procedural moments [6, 7]. Recent randomized trials have shown that GA may result in higher rates of successful recanalization compared to conscious sedation, local anesthesia, or monitored anesthesia care [6–11]. Furthermore, patients treated with GA may experience improved functional outcomes and a lower likelihood of experiencing symptomatic intracranial hemorrhage [8, 11–13].

However, there is uncertainty about whether VA or TIVA should be used in the EVT procedure of AIS patients. Crimmins et al., Diprose et al., and Vinay et al. have found advantages in using TIVA instead of VA in this condition [14–16]. Controversially, Sivasankar et al. have suggested some benefits of using VA as an alternative to TIVA for this population [17]. To address this controversy, we conducted a systematic review and meta‐analysis to assess the optimal technique of GA for patients with AIS undergoing EVT.

## 2. Materials and Methods

This systematic review and meta‐analysis were executed and described in accordance with the Cochrane Collaboration Handbook for Systematic Reviews of Interventions and with the Preferred Reporting Items for Systematic Reviews and Meta‐Analyses (PRISMA) statement guidelines of the Enhancing the Quality and Transparency of Health Research (EQUATOR) Network [18].

### 2.1. Eligibility Criteria

This meta‐analysis includes studies that met all the following eligibility criteria: (1) studies comparing TIVA with VA; (2) enrolling patients who underwent EVT for AIS; and (3) no follow‐up restrictions. We excluded studies (1) that considered control and intervention as the same group or (2) with insufficient data about control/intervention. Supporting Table 1 outlines the anesthetic protocols used across studies, specifying which volatile or intravenous agents were applied.

We included adult patients with AIS undergoing EVT under GA. The intervention group received TIVA, defined as continuous infusions of propofol and remifentanil. The comparator group received VA, which consisted of sevoflurane in three studies and isoflurane in one. Primary outcomes were favorable functional outcome (defined as the Modified Rankin Scale [mRS] 0–2), mortality, successful recanalization (modified treatment in cerebral infarction [mTICI] 2b/3—indicating reperfusion of greater than 50%), and mean arterial pressure (MAP) during the procedure. All included studies were observational cohort studies.

### 2.2. Search Strategy

We systematically searched PubMed, Embase, and the Cochrane Central Register of Controlled Trials from October 2023 to February 2024 with the following search terms: “thrombectomy,” “intravenous OR TIVA,” and “volatile OR inhalational.” The references from all included studies were also searched manually for any additional studies. Two authors (M.G. and L.B.) independently extracted the data following predefined search criteria. Gray literature was excluded due to concerns regarding methodological heterogeneity and limited reporting transparency. The prospective meta‐analysis protocol was registered on PROSPERO in February 2024 under protocol CRD42024518910.

### 2.3. Endpoints and Subgroup Analyses

The outcomes were (1) favorable functional outcome for overall patients based on the mRS score of 0–2, corresponding to minimal or no disability, (2) overall mortality, (3) mean of MAP during the procedure, and (4) successful recanalization, evaluated by the mTICI score 2b/3. We performed subgroup analyses using data from adjusted odds ratio (aOR) that included (1) mortality and favorable functional outcome for overall patients, (2) favorable functional outcome in patients with anterior stroke, and (3) mortality and favorable functional outcome in patients with anterior stroke after 3 months of the event [19, 20]. For outcomes reported with aORs, we pooled the most fully adjusted models reported in each study. The covariates included in these models varied slightly but consistently accounted for key confounders such as age, NIHSS, and tPA use. Supporting Table 2 summarizes the covariates used in the adjusted analyses and the modeling approach in each study.

### 2.4. Quality Assessment

We assessed the risk of bias in nonrandomized studies by utilizing the Risk of Bias in Non‐Randomized Studies of Interventions (ROBINS‐I) tool [21]. Two separate authors (M.G. and L.B.) carried out the assessment of risk of bias. Any disagreements were addressed through discussion, and differences were resolved by reaching a consensus.

### 2.5. Statistical Analysis

An odds ratio (OR) with a 95% confidence interval was used to compare treatment effects for categorical endpoints. Continuous outcomes were compared with standardized mean differences (SMDs). Statistical significance was defined as *p* < 0.05. We assessed heterogeneity with *I* ^2^ statistics and the Cochran Q test; *p* values > 0.10 and *I* ^2^ > 40% were considered significant for heterogeneity. Given the observed variability across studies, we used a DerSimonian and Laird random‐effects model to account for both within‐ and between‐study heterogeneity. This approach was applied to both low and high heterogeneity outcomes, in accordance with Cochrane guidelines for meta‐analyses with small numbers of included studies.

We performed two sensitivity analyses by (1) removing each study from the outcome assessment called a leave‐one‐out analysis and (2) using aOR data, when available. All statistical analyses was performed using RStudio 4.3.3 with the packages meta, dmetar, metaphor, remotes, readr, readxl, ggplot2, tidyverse, grid, and devtools.

Certainty of evidence was rated, at the outcome level, using the Grading of Recommendations Assessment, Development, and Evaluation (GRADE) approach. We followed the guidance developed by the Cochrane GRADEing Methods Group and the Cochrane Statistical Methods Group. Given that our review was limited to observational studies, the baseline GRADE rating for each outcome was “low.” This was adjusted for inconsistency, imprecision, and indirectness [22].

## 3. Results

### 3.1. Study Selection and Baseline Characteristics

**FIGURE 1:**
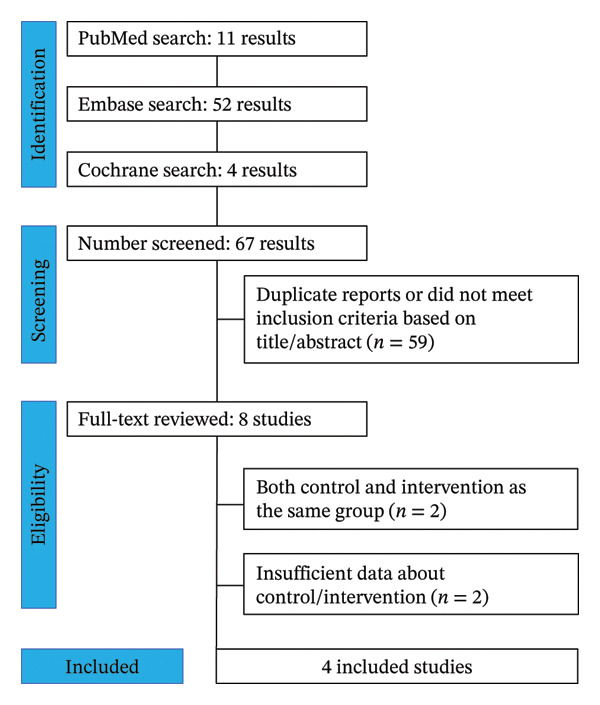
PRISMA flow diagram of study screening and selection.

As detailed in Figure 1, the initial search yielded 67 studies. After removing duplicate and ineligible studies, eight remained and were thoroughly reviewed based on the inclusion criteria. We intentionally restricted our search to peer‐reviewed articles indexed in PubMed, Embase, and Cochrane CENTRAL. This approach is in accordance with best practices specified in the Cochrane Handbook, which recommends excluding gray literature when methodological consistency and transparency are prioritized in smaller‐scale meta‐analyses. Considering that our review consists of just four observational cohort studies, the integration of lower‐quality or unpublished data could have added more inconsistency than benefit. Our objective was to incorporate studies that evaluated outcomes using standardized metrics (such as mRS, mTICI, and MAP) and showed methodological transparency. Gray literature often lacks detailed statistical data, peer review, or formal quality assessment, which would limit our ability to apply tools such as ROBINS‐I and GRADE effectively. As noted in our manuscript, with fewer than 10 studies, tests for publication bias (e.g., funnel plots and Egger’s test) are unreliable. While publication bias cannot be excluded, our analytical approach—particularly the use of aORs—helps mitigate some of this risk.

Ultimately, four cohort studies comprising 568 patients were included. A total of 381 (67%) patients received VA, and 187 (33%) patients received TIVA. Baseline characteristics of the patients are presented in Table 1. For patients who received VA, a total of 193 (50%) patients had intravenous tPA before EVT, and for those who received TIVA, 83 (44%) patients had tPA before the procedure. As seen in Table 1, the median (IQR) NIHSS was 19 [15–23] for VA and 21.5 [18–25] for TIVA, indicating slightly higher baseline stroke severity in the TIVA group.

**TABLE 1:** Baseline characteristics of included studies.

Study	No of patients volatile/IV	Baseline MAP (mmHg) volatile/IV	Age mean (y) volatile/IV	Sex (male), volatile/IV	Hypertension, volatile/IV	AFib, volatile/IV	DM, volatile/IV	Dyslipidemia, volatile/IV	NIHSS ‐ median (IQR), volatile/IV	IV tPA, volatile/IV
Diprose et al., 2019	254/59	—	64.4/66.2	57.9/59.3	64.6/69.5	47.2/52.5	16.5/23.7	42.9/44.1	16/18	58.3/37.3
Vinay et al., 2023	51/64	106.9/102.2	62.9/57.1	72.5/62.5	68.6/70.3	29.4/34.4	54.9/39.1	76.5/65.6	18/20	37.4/22.6
Sivasankar et al., 2015	35/12	109.5/102.0	62.7/69.8	62.8/41.6	77.1/91.6	57.1/58.3	25.7/41.6	60/75	19/21.5	37.1/58.3
Crimmins et al., 2022	41/52	102.4/105.5	66/66.5	63.4/51.9	46.3/59.6	26.8/28.8	12.2/23.1	19.5/28.8	18/16.5	36.6/53.8

*Note:* Unless noted otherwise, data are %. IV tPA: intravenous tissue‐type plasminogen activator; AFib: atrial fibrillation.

Abbreviations: DM, diabetes mellitus; IQR, interquartile range; MAP, mean arterial pressure; NIHSS, National Institute of Health Stroke Scale.

### 3.2. Pooled Analysis of All Studies

In unadjusted analyses, no significant differences were found between TIVA and VA for favorable functional outcome (OR 1.28; 95% CI [0.72; 2.27]; *p* = 0.403; *I* ^2^ = 50%) (Figure 2(a)); mortality (OR 0.54; 95% CI [0.27; 1.08]; *p* = 0.082; *I* ^2^ = 44%) (Figure 2(b)); mean MAP during the procedure (MD −0.5568; 95% CI [−4.0603; 2.9467]; *p* = 0.755; *I* ^2^ = 45%) (Figure 2(c)); and successful recanalization mTICI 2b/3 (OR 1.38; 95% CI [0.71; 2.67]; *p* = 0.344; *I* ^2^ = 0%) (Figure 2(d)).

FIGURE 2Analysis using raw numbers demonstrating overall favorable functional outcome (a), overall mortality (b), mean MAP during the procedure (c), and successful recanalization mTICI 2b/3 (d).

**Figure:**
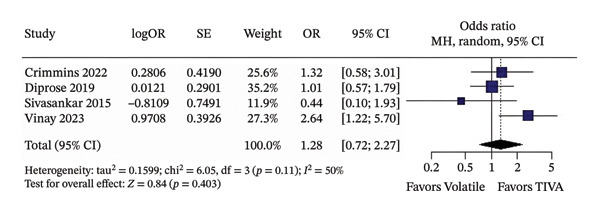
(a)

**Figure:**
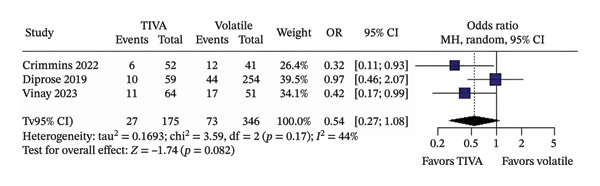
(b)

**Figure:**
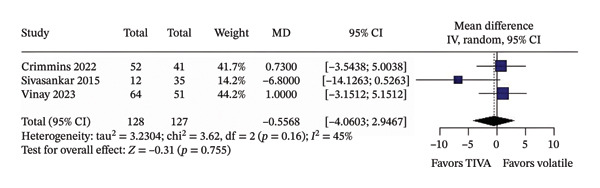
(c)

**Figure:**
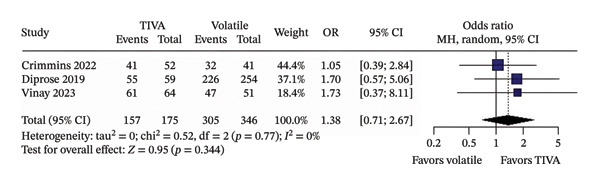
(d)

Interestingly, the TIVA group exhibited higher initial NIHSS scores and a lower incidence of intravenous thrombolysis compared to the VA group (Table 1), both of which are typically linked to poorer outcomes. Nevertheless, adjusted analyses consistently favored TIVA, indicating a potentially significant therapeutic effect. aORs provide a more precise representation of the actual influence of the anesthetic method by considering essential confounders, such as stroke severity, age, and pretreatment factors. Conversely, unadjusted analyses might produce misleading links due to baseline imbalances. The continued presence of positive results with TIVA in adjusted models, despite negative baseline characteristics, strengthens the theory that TIVA may be linked to better functional outcomes in this group.

**FIGURE 3:**
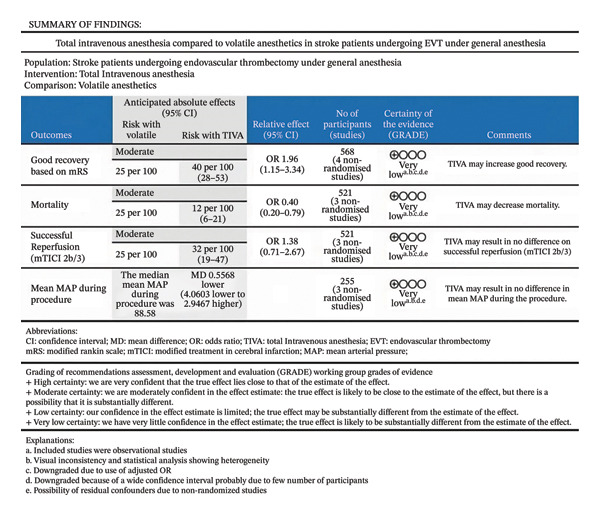
GRADE summary of findings for total intravenous anesthesia versus volatile anesthesia in stroke patients undergoing endovascular thrombectomy under general anesthesia, including estimates of effect size, absolute and relative risks, and certainty of the evidence.

Figure 3 outlines the GRADE quality of evidence evaluation for favorable functional outcome, mortality, successful reperfusion, and mean MAP during the procedure. Due to serious imprecision, indirectness, and inconsistency, we considered the overall quality of evidence to be “very low” for all outcomes.

### 3.3. Subgroup Analysis

When the aOR data were analyzed for overall patients receiving TIVA, it showed that TIVA probably increases favorable functional outcome (OR 1.96; 95% CI [1.15; 3.34]; *p* = 0.013; *I* ^2^ = 23%) and probably reduces mortality (OR 0.40; 95% CI [0.20; 0.79]; *p* = 0.008; *I* ^2^ = 0%) when compared with VA (Figure 4).

FIGURE 4Analysis using adjusted OR data for overall patients demonstrating favorable functional outcome (a) and mortality (b).

**Figure:**
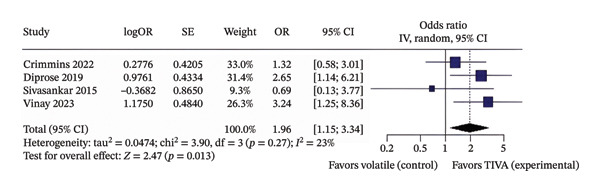
(a)

**Figure:**
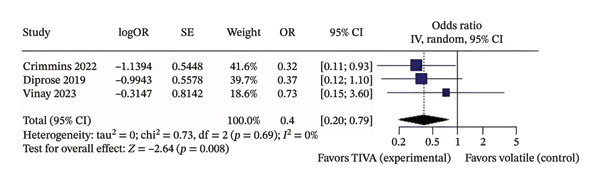
(b)

The discrepancy between unadjusted (Figures 2(a) and 2(b)) and adjusted (Figures 4(a) and 4(b)) ORs for Vinay 2023 reflects the effect of controlling for confounders such as stroke location and MAP. Adjusted estimates better represent the effect of anesthesia modality. We also confirmed that Sivasankar et al. explicitly included only anterior circulation strokes in their Methods section.

A subanalysis was performed for patients with anterior circulation stroke using data from aOR. When studies did not report outcomes at a fixed follow‐up interval, the effect of TIVA was not statistically significant (OR 2.15; 95% CI [0.93; 4.96]; *p* = 0.074; *I* ^2^ = 54%) (Figure 5(a)). Another subanalysis was made because 3 of the 4 studies evaluated the patients specifically after 3 months of the stroke. The patients who received TIVA and were assessed after 3 months showed a probable increase in favorable functional outcome (OR 2.66; 95% CI [1.08; 6.55]; *p* = 0.033; *I* ^2^ = 58%) (Figure 5(b)) and probably lower mortality (OR 0.38; 95% CI [0.20; 0.70]; *p* = 0.002; *I* ^2^ = 0%) (Figure 5) than patients who received VA.

FIGURE 5Analysis using adjusted OR data for anterior stroke patients demonstrating overall good recovery (a), favorable functional outcome based on mRS after 3 months (b), and mortality after 3 months (c).

**Figure:**
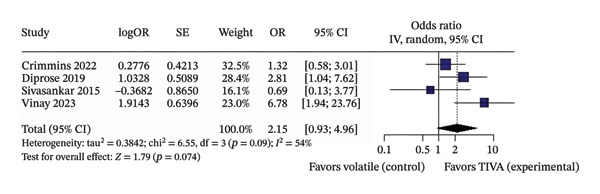
(a)

**Figure:**
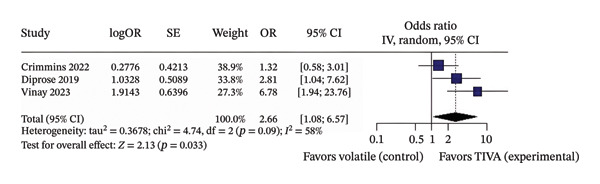
(b)

**Figure:**
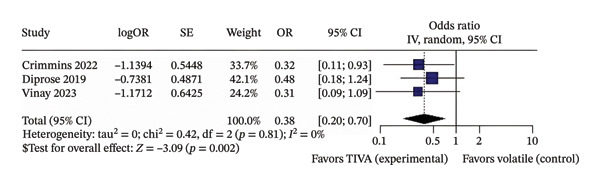
(c)

Observed statistical heterogeneity may be partially explained by center‐specific anesthetic practices, differences in hemodynamic targets, and variation in baseline stroke severity. For instance, TIVA groups had higher median NIHSS and lower tPA use, which would be expected to worsen outcomes, yet they performed better. This paradox strengthens the adjusted results. Random‐effects estimates were calculated using the DerSimonian and Laird method to account for both within‐ and between‐study heterogeneity.

A leave‐one‐out sensitivity analysis was conducted for findings that possibly reject the null hypothesis of no effect based on data from aOR. For favorable functional outcome in overall patients, the omission of Study 1 (Crimmins 2022) seems to have a relatively more considerable influence on the estimation of the overall effect size compared with other studies (Figure 6(a)). Omitting Study 1 causes the overall OR to increase by roughly 0.47. In contrast, mortality conclusions were not considerably changed in this analysis (Figure 6(b)).

FIGURE 6Leave‐one‐out analysis using data from adjusted OR for favorable functional outcome (a) and mortality (b) in overall patients and favorable functional outcome (c) and mortality after 3 months (d) for anterior stroke patients.

**Figure:**
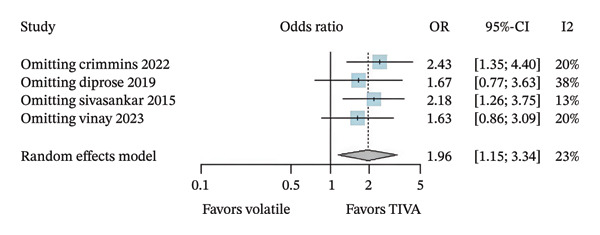
(a)

**Figure:**
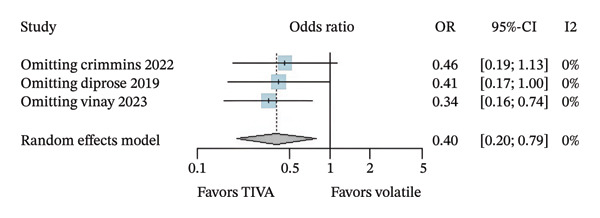
(b)

**Figure:**
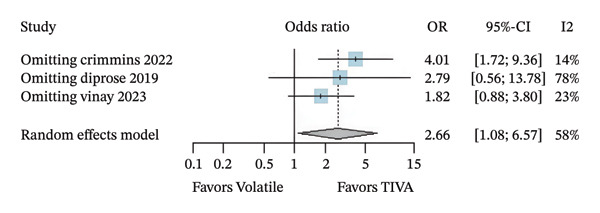
(c)

**Figure:**
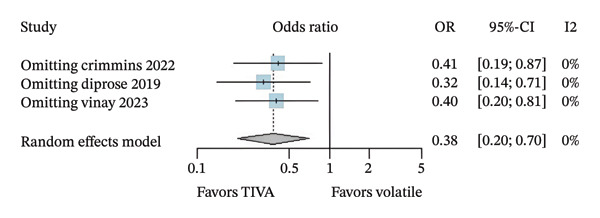
(d)

The absolute difference in favorable outcomes suggests a potential benefit: For every 100 patients treated with TIVA, approximately 15 more may achieve favorable functional outcome, and 13 fewer may die compared to VA. These clinically meaningful differences highlight the urgent need for a large, well‐powered randomized controlled trial to confirm these observations and establish causality.

Regarding anterior stroke patients, the omission of Study 1 (Crimmins 2022) seems to have a similar influence as before, showing a possibly larger influence once its omission causes the overall OR to increase by roughly 1.35 (Figure 6(c)). Mortality conclusions maintained that the analysis has not changed considerably (Figure 6(d)).

### 3.4. Quality Assessment

All four studies were considered to have a serious risk of bias, as described in Table 2. We did not include a funnel plot and an Egger test since the assessment of funnel plots has restricted value when the sample size is small, and the Egger test is not suggested until at least 10 studies are summarized [21]. A table including a summary of findings is shown in Figure 3. It includes results found with raw numbers.

**TABLE 2:** Risk of bias summary for non‐randomized studies.

Study	Bias due to confounding	Bias in the selection of participants in the study	Bias in the classification of interventions	Bias due to deviations from intended intervention	Bias due to missing data	Bias in the measurement of outcomes	Bias in the selection of the reported result
Crimmins et al., 2022	Serious	Low	Low	Low	Low	Low	Low
Diprose et al., 2019	Serious	Low	Low	Low	Low	Low	Low
Sivasankar et al., 2015	Serious	Low	Low	Low	Low	Low	Low
Vinay et al., 2023	Serious	Low	Low	Low	Serious	Low	Low

## 4. Discussion

This meta‐analysis of 568 patients from four observational studies suggests that TIVA may be associated with favorable functional outcome (mRS 0–2) and reduced mortality at 3 months, particularly in anterior circulation strokes. These associations remained consistent across adjusted and sensitivity analyses, despite baseline imbalances such as higher NIHSS and lower tPA use in the TIVA group.

The observational nature of the studies, with moderate heterogeneity, introduces a serious risk of bias, including confounding by indication and selection bias. Notably, despite worse baseline characteristics, the TIVA group showed better outcomes, suggesting a potential benefit that warrants further investigation. This consistent direction of effect in adjusted analyses across multiple domains suggests a clinically relevant association and also supports the hypothesis‐generating characteristic of this study.

Sivasankar et al. have suggested that VA may improve favorable functional outcome and brain tissue oxygenation in patients during EVT [17]. Preclinical studies have shown that VA, such as isoflurane, has neuroprotective properties in cases of ischemia [23]. VA can increase blood flow to the brain and has neuroprotective properties, but higher doses of sevoflurane can cause an uncoupling of cerebral flow and metabolism [15, 24]. Increasing doses of VA higher than 1.5 minimum alveolar concentration create augmented cerebral blood flow via direct vasodilatation. At the same time, metabolism declines, suggesting an impairment of cerebral autoregulation, which may lead to a reduction in cerebral blood flow to the ischemic area. This mechanism is known as the intracranial steal phenomenon, which can be detrimental in cases of AIS [16, 24].

On the other hand, TIVA (propofol–remifentanil) reduces cerebral blood flow and metabolism, but it maintains the coupling of cerebral flow–metabolism even in a state of profound anesthesia [24]. The reduction in metabolism, which is more significant than the reduction in flow, leads to indirect vasoconstrictive properties [24, 25]. This increased resistance in a low‐flow setting increases autoregulation, while VA’s intrinsic tendency to reduce cerebral resistance has the opposite effect [24]. Several preclinical trials have suggested that propofol, widely used in TIVA, protects brain cells after injury, making it an effective strategy for neuroprotection in stroke [26]. Proposed mechanisms for propofol’s neuroprotection include antiapoptotic, anti‐inflammatory, and antioxidative effects, as well as reduced infarct size and blood–brain barrier preservation [27–34]. A recent preclinical study compared the magnetic resonance imaging changes after middle cerebral artery occlusion when using TIVA versus VA. The study found that rats under TIVA had a reduced final infarct size and functional deficit, less cytotoxic and vasogenic edema, reduced blood–brain barrier disruption, and preserved cerebrovascular reactivity compared to isoflurane [35].

Blood pressure control is critical for good outcomes. GA predisposes patients to a drop in blood pressure, an imperative factor to be managed in patients undergoing EVT for AIS [17, 36]. Episodes of low blood pressure have been linked to poor functional outcomes. At the same time, high blood pressure can also be harmful. Rasmussen et al. have detected an association of poor outcomes in patients presenting with MAP less than 70 mmHg for more than 10 min and MAP higher than 90 mmHg for more than 45 min [37, 38]. However, the best anesthesia technique for maintaining blood pressure remains debatable. Sneyd et al. found increased hypotensive episodes in the sevoflurane group compared to propofol, while Chauhan et al. showed the opposite [36, 39]. Controversially, Vinay et al. and Crimmins et al. observed no difference in mean MAP between propofol and sevoflurane [14, 16]. Similarly, our findings did not suggest a difference in mean MAP during the procedure between both GA techniques.

The choice between VA and TIVA also affects other factors like environmental impact and drug delivery systems. VA agents require precise titration and are influenced by intraoperative variables, which can potentially complicate cerebral perfusion [40, 41]. TIVA, delivered via target‐controlled infusion, allows more stable hemodynamic control [42]. Environmentally, volatile agents contribute significantly to greenhouse gas emissions [43, 44], while propofol, used in TIVA, is hepatically metabolized and poses minimal ecological risk [45]. As sustainability becomes a healthcare priority, both physiologic and environmental factors should inform anesthetic choice during EVT.

Clinically, current anesthetic practices vary significantly worldwide. In North America and Europe, both TIVA and VA are used with no consensus, while in South America, resource availability and training often favor VA. Some centers in Brazil and Argentina use primarily inhalational anesthesia due to limited access to infusion pumps or target‐controlled infusion systems, whereas others are increasingly adopting TIVA protocols [46–49]. This heterogeneity underscores the importance of identifying the most effective approach.

Our study has some significant limitations that we need to consider. Few observational published studies have compared TIVA to VA in patients with AIS undergoing EVT. There are no randomized studies in this field. This highlights a key research gap in optimizing stroke anesthesia care. To mitigate the limitation of nonrandomized studies, we have made the best use of the available data using aOR and leave‐one‐out analyses. These methods strengthen internal validity, although residual confounding remains possible. Furthermore, we found significant heterogeneity in the favorable functional outcome group for patients with anterior circulation stroke, and to minimize such heterogeneity, we performed a leave‐one‐out analysis.

Given the potential for improved neurological outcomes and mortality reduction with TIVA, along with favorable physiologic and environmental profiles, centers equipped with intravenous anesthesia infrastructure may consider TIVA as a preferred strategy for EVT in AIS. However, these findings must be confirmed through well‐powered, prospective randomized trials before practice guidelines can be revised.

## 5. Conclusion

Our findings suggest that TIVA may be connected to better functional outcomes and lower death rates, especially in patients who have strokes in the anterior circulation. When examining pooled, unadjusted data, no statistically significant differences were observed. However, when examining adjusted models, TIVA consistently outperformed VA, with higher chances of achieving a favorable functional outcome (mRS 0–2) and lower mortality rates despite baseline imbalances. The groups seemed to have similar levels of hemodynamic stability and rates of successful recanalization, though wide confidence ranges reflect persistent uncertainty regarding differential effects. Although all included studies had a high risk of bias and a very low level of evidence according to GRADE criteria, the consistent findings across diverse datasets suggest a biologically and clinically reasonable suggestion of a potentially meaningful link in favor of TIVA.

Several limitations of this study must be acknowledged. First, all included studies were observational cohort studies and were therefore judged to have a serious risk of bias due to confounding, as assessed by the ROBINS‐I tool. This design inherently limits causal inference and introduces the potential for residual confounding despite multivariable adjustment and propensity score–based analyses. Second, the number of available studies and the overall sample size were small, reflecting the limited published evidence directly comparing VA and TIVA during EVT. This contributed to serious imprecision and inconsistency, which led to the overall very low certainty of evidence for all outcomes according to GRADE criteria. Third, heterogeneity across studies (including differences in patient selection, stroke severity, anesthetic protocols, hemodynamic management, follow‐up timing, and covariates included in adjusted models) may further limit the generalizability of our findings.

Although we attempted to mitigate these limitations through the use of aORs, random‐effects modeling, and leave‐one‐out sensitivity analyses, these approaches cannot fully eliminate bias. Consequently, the results should be interpreted as hypothesis‐generating and highlight the need for well‐designed, prospective, multicenter randomized trials. In the meantime, TIVA might be tentatively considered a potentially advantageous approach in this scenario.

**Nomenclature**
EVT: Endovascular thrombectomy
AIS: Acute ischemic stroke
VA: Volatile anesthesia
TIVA: Total intravenous anesthesia
GA: General anesthesia
mRS: Modified Rankin Scale
mTICI: Modified treatment in cerebral infarction
MAP: Mean arterial pressure

## Funding

No funding was received for this manuscript.

## Conflicts of Interest

The authors declare no conflicts of interest.

## Supporting Information

Additional supporting information can be found online in the Supporting Information section.

## Supporting information


**Supporting Information** Supporting Table 1: Anesthetic protocols. Supporting Table 2: Adjusted covariates in primary studies.

## Data Availability

The data used to support the findings of this study are included within the article.
