# Supplementary material for: Comparison of Volatile Anesthesia and Intravenous Anesthesia for Endovascular Thrombectomy in Acute Ischemic Stroke Patients Under General Anesthesia: A Systematic Review and Meta‐Analysis
Source: Crit Care Res Pract. 2026 May 28;2026:7360200. doi: 10.1155/ccrp/7360200 (PMC13218072; doi:10.1155/ccrp/7360200)
Supplement: Supplementary file 1 — Supporting Information Supporting Table 1: Anesthetic protocols. Supporting Table 2: Adjusted covariates in primary studies. [file CCRP-2026-7360200-s001.docx]

| **Supplementary TABLE 1 - Anesthetic Protocols** | | | |
| --- | --- | --- | --- |
| **Study** | TIVA PROTOCOL | VA PROTOCOL | NOTES |
| Crimmins et al., 2022 | Propofol + Remifentanil | Sevoflurane | TIVA via TCI system; BIS-guided |
| Diprose et al., 2019 | Propofol + Remifentanil | Sevoflurane | Balanced Anesthesia; BIS monitoring |
| Sivasankar et al., 2015 | Propofol + Remifentanil | Sevoflurane | Remifentanil infusion titrated to effect |
| Vinay et al., 2023 | Propofol + Remifentanil | Isoflurane | Older protocol, limited data, institutional |

| **Supplementary TABLE 2 - Adjusted Covariates in Primary Studies** | | |
| --- | --- | --- |
| **Study** | Covariates included | Model Type |
| Crimmins et al., 2022 | Age, sex, NIHSS, tPA use, comorbidities (HTN, DM), onset-to-puncture time | Multivariable logistic regression |
| Diprose et al., 2019 | NIHSS, tPA use, age, procedure duration | Multivariable logistic regression |
| Vinay et al., 2023 | Age, sex, NIHSS, stroke location, tPA use | Propensity score-adjusted logistic regression |
